# Reconstruction with a Custom-made 3D-printed Prosthesis for Limb Salvage and Joint Preservation after Resection of the Calf Myxofibrosarcoma Involving the Tibia: A Case Report and Literature Review

**DOI:** 10.2174/0115734056415924251022041623

**Published:** 2025-10-29

**Authors:** Haoyuan Du, Ping Wang, Qi Liu, Zhiping Deng, Weifeng Liu, Jie Zhao, Junqiang Wang

**Affiliations:** 1 Department of Orthopaedic Surgery, Beijing Jishuitan Hospital, Capital Medical University, Beijing, 100035, China; 2 Department of Orthopaedic Surgery, The Affiliated Hospital of Shandong University of Traditional Chinese Medicine, Jinan, Shandong 250014, China; 3 Department of Radiology, Beijing Jishuitan Hospital, Capital Medical University, Beijing, 100035, China; 4 Department of Orthopaedic Oncology Surgery, Beijing Jishuitan Hospital, Capital Medical University, Beijing, 100035, China

**Keywords:** Calf myxofibrosarcoma, 3D-printed prosthesis, Critical-sized tibia defects, Soft tissue tumor, Bone invasion, Limb salvage, Joint preservation

## Abstract

**Introduction:**

Myxofibrosarcoma (MFS) is a rare form of soft tissue sarcoma, distinguished by its local aggressiveness and a high recurrence rate. However, cases involving the lower leg and progressively affecting large segments of the tibia are infrequently reported in the literature. In this study, we report the case of a 76-year-old patient with MFS of the right calf, which progressively affected critical-sized tibial defects. The patient underwent surgical resection followed by reconstruction using an individualized three-dimensional (3D)-printed prosthesis, which preserved both the knee and ankle joints.

**Case Presentation:**

This report describes the case of a 76-year-old Chinese woman with MFS of the right calf, which progressively invaded the mid-tibia. She underwent wide tumor resection and reconstruction using a customized 3D-printed prosthesis that preserved both the tibial epiphysis and ankle joint. The procedure successfully preserved the entire articular surface, providing a viable alternative for maintaining both the function and integrity of the affected limb. The patient maintained normal knee joint function, with no evidence of recurrence or metastasis observed during the 2-month postoperative follow-up.

**Conclusion:**

Reconstruction of critical-sized tibial defects with a customized 3D-printed non-cemented prosthesis after resection of a large MFS tumor demonstrated excellent mechanical stability during the early follow-up period in this knee and ankle joint-preserving procedure. Further investigation into the durability and long-term complication rates is necessary before this approach can be incorporated into routine clinical practice.

## 1 INTRODUCTION

Myxofibrosarcoma (MFS) is a relatively rare form of soft tissue sarcoma, typically arising in the subcutaneous connective tissue. It accounts for approximately 5-10% of all soft tissue sarcomas, with the majority of cases occurring in individuals between the ages of 50 and 80, without significant gender differences in incidence [1]. The lower limbs, particularly the thigh, are the most commonly affected sites. Due to its tendency for subcutaneous tissue invasion, MFS is highly aggressive and prone to recurrence. While the risk of distant metastasis is relatively low, lung metastasis may occur in cases of disease progression or high-grade MFS [2]. Previous studies have indicated that MFS predominantly affects the soft tissues of the lower extremities, with only a few cases involving large segments of the tibia in the lower leg [3-7]. In a report by Wakely *et al*., 66 cases of MFS were analyzed, with only 5 tumors exceeding 15 cm in length, and the largest measuring 23 cm [8]. Of these, just 6 cases involved the lower leg, with an average invasion length of 8.6 cm. In contrast, we report a case of MFS originating in the soft tissue of the lateral anterior tibial muscle, which invaded large segments of the tibia within 4 months, reaching a maximum invasion length of 29 cm. Treatment involved wide resection of the tumor, followed by reconstruction using a custom-made 3D-printed joint-preserving prosthesis.

Advances in technology, such as artificial intelligence (AI), 3D preoperative planning, and 3D printing, have revolu-tionized the management of complex bone tumor cases. AI and machine learning algorithms have shown great promise in preoperative imaging analysis, aiding in the identification and segmentation of tumors, predicting surgical outcomes, and customizing treatment plans [9-11]. Additionally, 3D preope-rative planning enables precise visualization of the tumor and surrounding anatomy, allowing surgeons to devise the most effective strategy for resection and reconstruction [12]. Moreover, the development of 3D-printed implants has trans-formed prosthesis customization, providing highly personalized solutions that optimize joint function and reduce complications [13, 14]. These technological advancements were integral to the successful reconstruction in the present case.

This study was approved by the Institutional Review Board of Affiliated Jishuitan Hospital, Capital Medical University, China. Informed consent for the procedure was obtained from the patient.

## 2 CASE REPORT

A 76-year-old Chinese woman presented to our hospital with a primary complaint of superficial and deep soreness in her right lower leg for the past 6 months, accompanied by noticeable swelling in the anterior lower limb. Initially, a small mass was detected in the anterior right calf. Despite a 6-month course of traditional Chinese treatments, including massage, acupuncture, and moxibustion, along with NSAIDs, the swelling progressively worsened, and pain began to interfere with the patient's daily activities. Prior to surgery, a comprehensive preoperative evaluation was conducted, which included the following assessments: (1) range of motion (ROM): right knee flexion was limited to 10° to 100°; (2) visual analog scale (VAS): pain score was 8/10 at rest (severe pain) and 9/10 during movement (very severe pain); (3) muscle strength: normal strength was observed; (4) SF-36: the patient scored 40 for physical functioning and 45 for bodily pain.

**Fig. (1):**
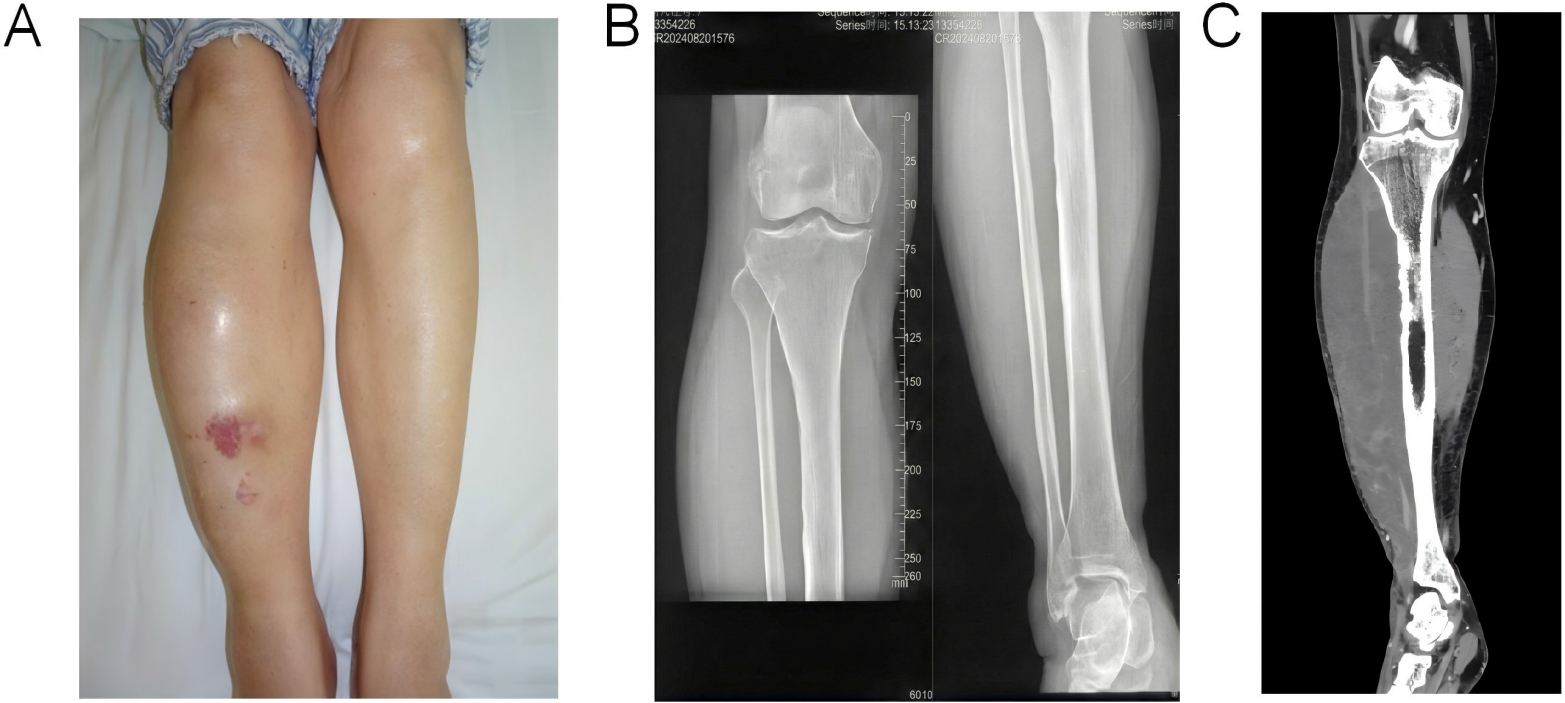
(**A**) Obvious swelling of the middle and lower right calf with local rash, lobulated nodules, and skin damage. (**B**) Anteroposterior (AP) and lateral X-ray images of the right tibia, showing an internal mass of the right tibia anterior muscle. (**C**) CT images of the right lower limb showing an extensive soft tissue tumor and tibial cortex involvement.

**Fig. (2):**
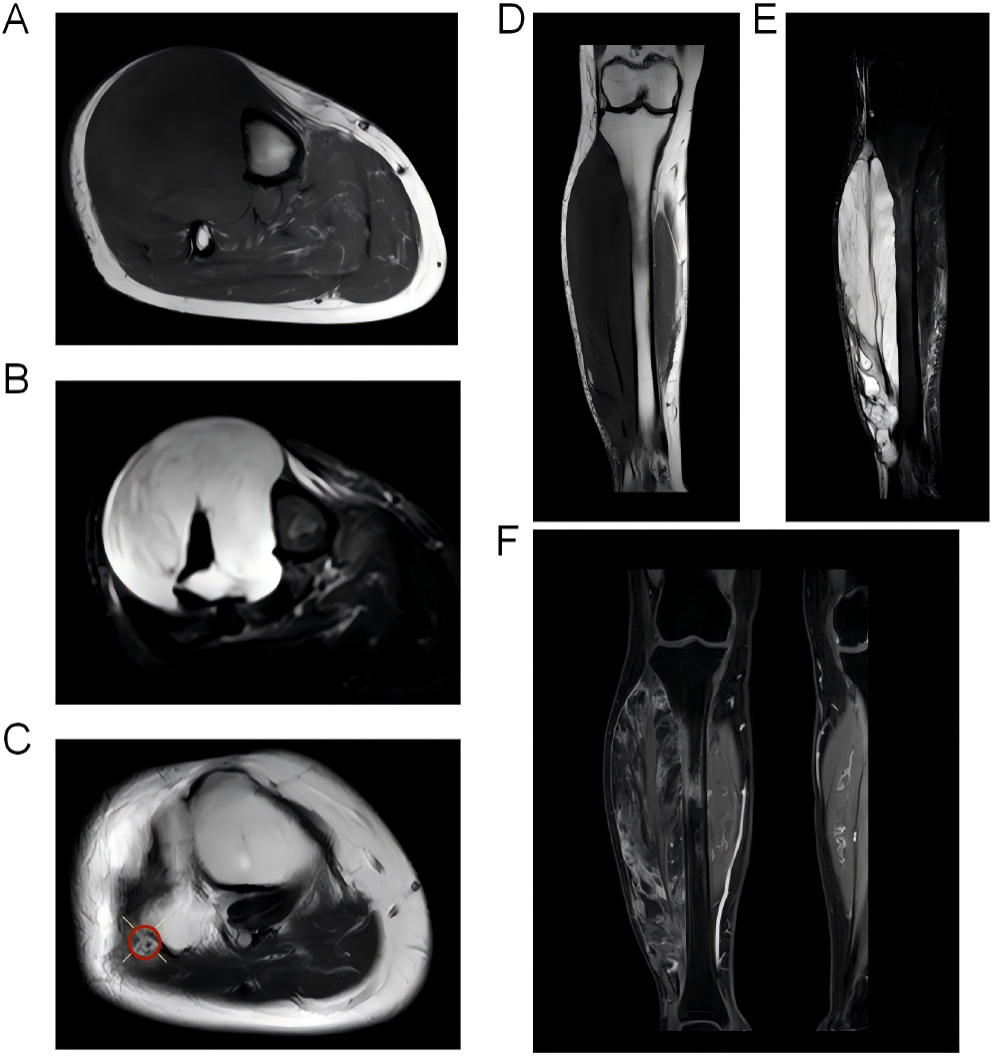
Preoperative MRI of the left calf showing an invasive lesion in the midstream tibia. The axial (**A**) T1-weighted image; (**B**) the “tail sign” can be clearly seen through the FS-weighted image; (**C**) T2-weighted image showing the common peroneal nerve to be invaded by the tumor at the location of the small head of the fibula. The coronal (**D**) T1-weighted, and (**E**) T2-weighted images. The sagittal (**F**) contrast-enhanced Dixon fat-water separation imaging.

Clinical examination revealed significant swelling in the right calf, more pronounced than the left. CT imaging showed invasion of the lateral cortical bone of the tibia, with an irregular surface (Fig. **1A**-**C**). MRI demonstrated an invasive lesion in the midstream tibia with osteolytic destruction measuring 7 cm x 6.3 cm x 29.4 cm. Mixed signals were observed in the mid-distal right tibia and distal fibula, with low and high signals on T1- and T2-weighted images, respectively (Fig. **2A**-**F**). The tumor was classified as Grade 3 MFS according to the WHO grading system, aligning with the AJCC stage III classification for soft tissue sarcomas. The patient's legal guardians were fully informed about the study protocol and provided consent for the publication of the case data.

**Fig. (3):**
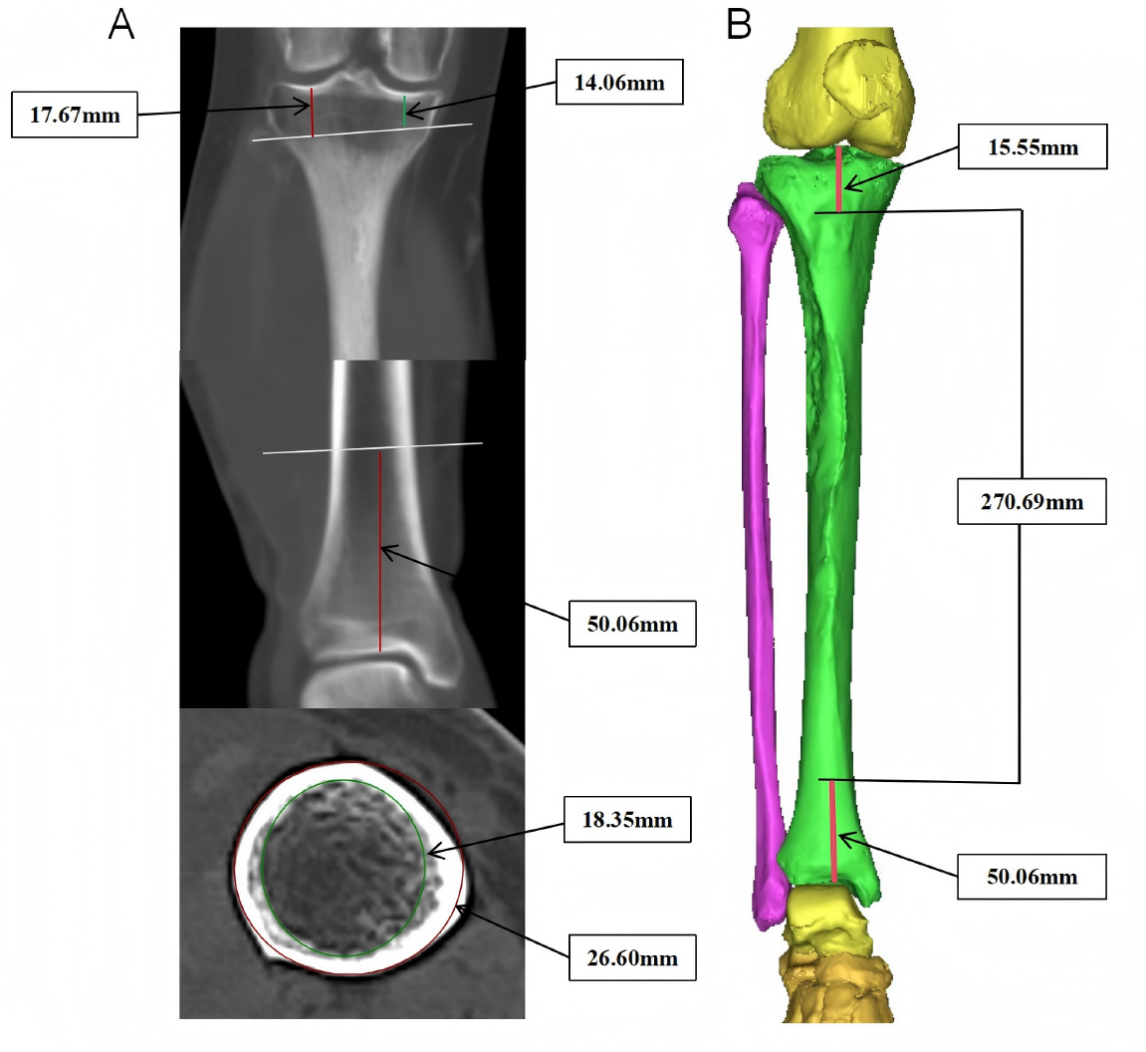
(**A**) The length of the osteotomy at the proximal part was 15.55 mm from the knee surface, and at the distal part, it was 50 mm from the ankle surface. The width of the reaming was also planned preoperatively. (**B**) The final osteotomy length and model were planned using 3D printing, with the overall osteotomy length measuring approximately 27.70 cm.

**Fig. (4):**
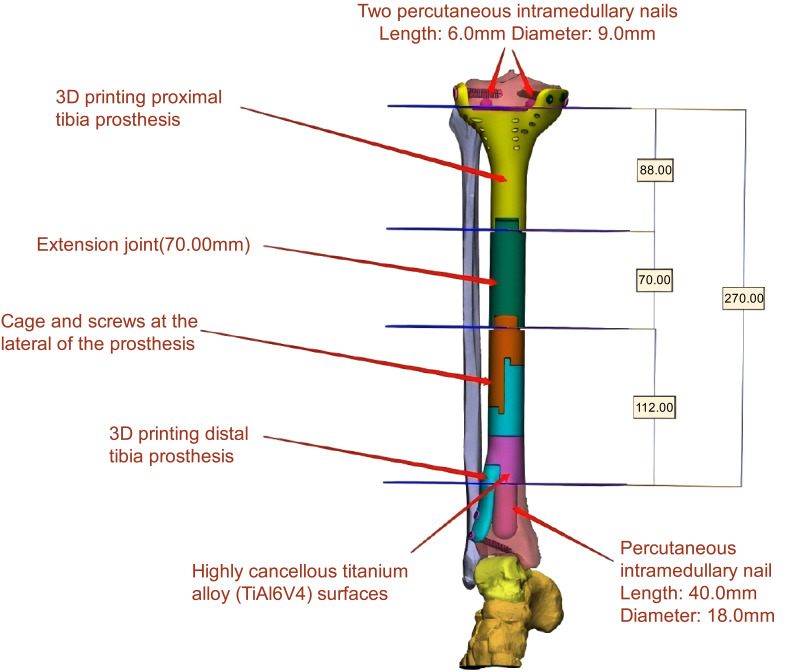
3D printing prosthesis design scheme and installation effect diagram.

The tumor tissue was partially located subcutaneously, exhibiting extensive longitudinal and invasive growth, which had already enveloped the common peroneal nerve, tibia, and anterior tibial vessels. Due to local recurrence and the invasion of peripheral nerves and blood vessels, above-knee amputation was considered a curative treatment option. However, the patient expressed a strong preference for limb-sparing surgery. Consequently, limb-sparing surgery was pursued, which involved the extensive excision of the tumor, the affected tibia, and the encapsulated common peroneal nerve and anterior tibial vessels, followed by the repair of the critical-sized bone defect using a custom-made 3D-printed tibial prosthesis. The location and extent of the tumor were simulated using 3D CT reconstructions and MRI data prior to surgery, and the osteotomy site was planned with a 3 cm safety margin. The proximal tibial osteotomy was positioned approximately 15 mm below the highest point of the intercondylar eminence of the tibial plateau, while the distal osteotomy was positioned about 50 mm above the lowest point of the ankle mortise, resulting in a total osteotomy length of approximately 270 mm. A 3D-printed porous prosthesis was designed for the reconstruction of large tibial bone defects following tumor resection (Figs. **3A**,**B** and **4**).

The prosthesis consisted of proximal and distal 3D-printed tibial components connected by intermediate connective elements. The articular surface of the proximal tibia and the collateral ligaments were preserved. The contact surface between the prosthesis and the host bone was designed with a titania-sprayed trabecular bone structure. To ensure both mechanical stability and biological fixation, two medullary needles (9 mm in diameter, 6 mm in length) were used for intramedullary biological fixation, and three titanium-coated screws were installed on each side to provide initial stability. The trabecular bone mesh coating on both the proximal tibia component and distal tibia stem enhanced the bond between the prosthesis and the host bone, thereby eliminating the need for bone cement fixation. The ankle joint was preserved at the distal tibia, and the prosthesis stem was designed with a 40 mm length and 18 mm diameter, coated with trabecular bone mesh to further improve fixation. The use of titanium-coated screws reinforced the structural stability of the prosthesis, promoting long-term functional recovery and minimizing the risk of mechanical failure (Fig. **4**).

**Fig. (5):**
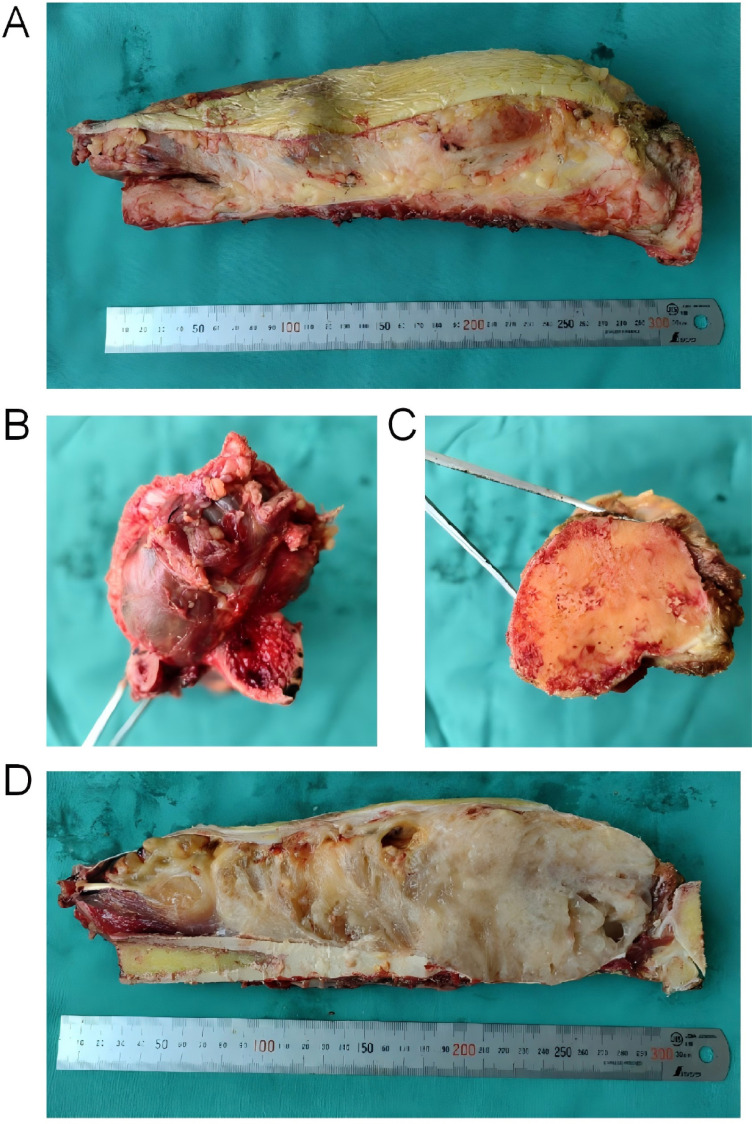
(**A**) Complete excision of the bone together with the tumor and the invaded tibia. (**B**) The gross tissue structure of the tumor. (**C**) At the axial section of the proximal tibia, the incisal margin was negative, with no tumor tissue identified. (**D**) The longitudinal section showed that the tumor tissue was mucus and had invaded most of the bone.

The patient was positioned supine. Following general endotracheal anesthesia, the surgical site was disinfected using iodine tincture and alcohol, and the procedure was performed under a tourniquet. A 35 cm incision was made, extending from the medial front of the calf to the medial border of the patella, and finally to the anterior malleolus. After incising the skin and subcutaneous tissue, the needle tract was completely excised in a fusiform fashion, and the flap was carefully elevated bilaterally. The tumor was clearly identified in the soft tissue of the anterior calf. The posterior tibial blood vessels and nerves were meticulously dissected and preserved, while the common peroneal nerve, anterior tibial blood vessels, and the tendons of the extensor digitorum longus and hallucis longus were severed due to complete tumor encasement. Using a preoperatively designed osteotomy guide, precise osteotomies were performed on the proximal and distal tibia. The tumor mass, along with a surrounding layer of normal tissue and the percutaneous biopsy tract, was completely excised (Fig. **5A**-**D**). The proximal prosthesis component was placed using a custom tool and secured with three screws on both the medial and lateral sides. A biotype prosthesis, with a lateral supplemental titanium plate, was expanded within the distal tibial canal and secured with two screws. After connecting the middle bone defect with a test module, the length of the lower limb was confirmed to be consistent. The assembly was completed, and the joint was secured with two bolts, while the distal tibia and fibula were bound using a titanium cable (Fig. **S1A**-**D**). The medial head of the gastrocnemius was repositioned to cover the prosthesis, and the patellar ligament and surrounding muscles were re-sutured with high-strength absorbable sutures. The dorsal extensor tendons of the foot (extensor digitorum longus and hallucis longus) were reinforced with surrounding muscles. The wound was thoroughly cleaned and sutured following adequate hemostasis. Prophylactic antibiotics were administered for 7 days, and a negative pressure drain was maintained until the drainage volume decreased to less than 20 mL/day. The ankle joint was immobilized with an external gypsum cast for 6-8 weeks. Isometric muscle exercises, as well as flexion and extension exercises of the knee joints, were initiated immediately after the operation.

Pathological examination of the resected specimen revealed invasive tumor growth under microscopic observation. Spindle-shaped tumor cells of varying density were observed within the mucoid stroma, distributed either sparsely or in dense sheets, exhibiting atypia with frequent pathological mitotic figures. MFS was diagnosed, with regions displaying low-to-moderate malignancy and others showing high-grade malignancy. Immunohistochemistry revealed the following results: SOX10 (-), H3K27me3 (+), S-100 (-), CD34 (partial +), desmin (-), SMA (-), Ki67 (index 70%), vimentin (+), CK (AE1/AE3) (partial +), and DDIT3 (-) (Fig. **S2A**,**B**).

The patient underwent routine follow-up at 2 weeks, 1 month, 2 months, 6 months, and 12 months postoperatively. Early assessments (2 weeks-2 months) showed stable prosthesis positioning, with no signs of infection, loosening, or recurrence. At 2 months, functional evaluation using the Musculoskeletal Tumor Society (MSTS) scoring system revealed a knee range of motion (ROM) of 10°-80° and an MSTS score of 28, indicating satisfactory early recovery. Radiographs at 6 and 12 months confirmed good integration of the prosthesis with the tibia, with no evidence of loosening or infection. Full-length lower-limb X-rays demonstrated restored alignment and nearly equal leg length. The surgical wound healed well, with no signs of redness, exudation, or complications. At 12 months, the patient achieved an ROM of 0°–110°, an MSTS score of 68, VAS scores of 1/10 at rest and 2/10 during movement, normal muscle strength, and significant improvement in SF-36 (physical functioning: 80, bodily pain: 30). Overall, the patient was fully satisfied (Fig. **S3**).

## 3 DISCUSSION

Myxofibrosarcoma (MFS) was first reported by Angervall *et al*. [15] in 1977. As a rare fibroblastogenic malignant soft tissue tumor, MFS is more commonly seen in the elderly, particularly in the extremities, followed by the trunk [16]. Clinically, MFS typically presents as raised, lobulated nodules on the skin, often accompanied by surrounding papular infiltrative lesions and pigmentation [17]. Radiographically, Robert *et al*. identified the “tail sign” in 52 pathologically confirmed cases of MFS on local MRI scans [18]. The high-signal mass on fat-suppressed T2-weighted images extends distally along the fascia and subcutaneous tissue in a linear and arc-shaped pattern. The tail sign has a sensitivity of 64-77% and a specificity of 79-90% for diagnosing soft tissue tumors, offering moderate sensitivity and specificity in identifying MFS compared to other myxoid tumors. Histopathologically, MFS displays a multinodular growth pattern at low magnification, with fibrous connective tissue septa between nodules and a myxoid stroma. At higher magnification, MFS cells are either disorganized or arranged in fascicles, with slender, curved capillaries and pseudolipoblasts present. A small amount of coagulative tumor necrosis is also observed [19, 20]. Compared to other soft tissue sarcomas (STSs), MFS is a distinct subtype known for its high recurrence rates and infiltrative nature. While the average recurrence rate for STSs is approximately 10%, the recurrence rate for MFS is notably higher, ranging from 14% to 61%. Cases involving the extremities are especially prone to local recurrence, and some patients with highly malignant forms may experience distant metastases [21]. Due to its invasive nature, MFS can progressively spread to surrounding bone.

Teresa *et al*. reported a case in which the primary lesion, initially located in the anterior soft tissue of the tibia, gradually expanded to involve the distal fibula, distal tibiofibular joint, and the lateral cortex of the distal tibia [3]. Similarly, Desai *et al*. described the case of a 40-year-old man who initially presented with mid-foot pain, which progressed over two months to affect the ipsilateral metatarsals [22]. We have summarized reported cases of MFS in the lower leg over the past 20 years (Table **1**). In our case, a similar infiltration pattern was observed. The patient initially presented with a soft tissue mass in the anterolateral region of the lower leg, which subsequently invaded the cortical bone of large segments of the tibia. Some scholars believe that adjuvant radiotherapy can help prevent local spread in many cases, while neoadjuvant radiotherapy (nRT) may be considered for some high-grade MFS [23]. In a systematic review of 10 studies from 301 relevant articles, Meagen *et al*. found that due to the infiltrative growth pattern of MFS, nRT is commonly used before surgery to improve local control [24]. Kamio *et al*. conducted a retrospective study analyzing records of 349 MFS patients from the nationwide Bone and Soft Tissue Tumor Registry in Japan (2006-2015), finding that adjuvant radiotherapy and chemotherapy did not significantly improve prognosis [25]. As a result, surgical resection remains the standard treatment for MFS [26-29]. Factors, such as tumor grade, size, and unplanned preoperative resections, can increase recurrence rates, with surgical margins playing a crucial role. Studies suggest that a minimum resection margin of at least 1 cm and a width of 2 cm can help reduce local recurrence rates [30]. Radical resection or amputation may be necessary in cases of rapid local recurrence and invasion [4, 21]. However, advances in multidisciplinary techniques, including imaging, computer navigation, 3D printing, chemotherapy, and surgical techniques, have increasingly favored limb-sparing surgery (LSS) as the preferred treatment for extremity sarcomas over recent decades. Even for tumors involving the metaphyseo-diaphyseal regions of long bones, segmental resection and reconstruction can preserve native joints.

Reconstruction methods for large segmental bone defects following tumor resection include both biological and mechanical options. Biological methods, such as allografts, distraction osteogenesis, vascularized fibula autografts, and recycled autografts, promote bone induction and conduction, but each has specific limitations. Mechanical options primarily involve custom-made prostheses. While bone allografts are often regarded as the “gold standard” for tumor reconstruction, they are difficult to obtain in large segments and carry significant risks of fracture, infection, and nonunion [31]. Vascularized fibula autografts (VFGs) have a smaller cross-sectional area, making them weaker than the original resected bone. These autografts are also more prone to fatigue fractures and complications at the donor site [31, 32]. Tumor-devitalized autografts (*e.g*., *in vitro* radiation inactivation, liquid nitrogen inactivation [33], microwave ablation, and hypertonic saline immersion inactivation) can lengthen operation times and often lead to complications, such as nonunion, delayed union, and early-stage weight-bearing difficulties for patients [34].

For large bone defects, 3D-printed custom prostheses offer notable advantages. In this case, the tumor extensively involved both soft and bone tissues. Following the strict “no tumor” excision protocol, at least 27 cm of the tibia needed to be removed. Zhao *et al*. defined an “ultra-critical-sized bone defect” as a tibial defect exceeding 15 cm, comprising over 60% of the tibia, or leaving a remaining proximal or distal tibial bone mass of just 0.5 to 4.0 cm [35]. Studies indicate that removing more than 60% of the tibia significantly lowers the success rate of traditional implants, such as allografts and vascularized fibula grafts (VFG), due to risks of secondary fractures and nonunion [36]. Therefore, 3D-printed prostheses are particularly effective in addressing such “ultra-critical-sized bone defects”. In a review of five lower limb reconstructions with 3D-printed prostheses, Zhao *et al*. reported an average defect length of 22.8 cm post-tumor resection, with an average residual bone length of 2.65 cm (range: 0.6–3.8 cm) [35]. All cases achieved successful biological fixation after a follow-up of 27.6 months. Considering that our patient was an elderly woman with slow bone regeneration, biological reconstruction could delay early weight-bearing and increase the risks of complications, such as thrombosis and hypostatic pneumonia. Therefore, we opted for mechanical reconstruction using 3D-printed guides to repair the tibial defect after extensive tumor resection (Table **2**).

Many scholars have also focused on hemicortical resection and biological reconstruction to preserve joint structure and bone tissue, thereby reducing trauma and improving postoperative limb function [37-40]. Wang *et al*. reported a case of medial ankle fibrosarcoma, where reconstruction involved 1/4 of the ankle joint [39]. At follow-up conducted 7 months and 3 years postoperatively, the patient exhibited satisfactory ankle function and stability, with no pain or complications. However, in most reported cases, bone defects ranged from 4 to 8 cm, with an average resection length of 7.8 cm, while defects larger than 8 cm were addressed only in a few instances [37-40]. In our case, the tumor had extensive longitudinal invasion of the tibia, necessitating a preoperative osteotomy of approximately 27 cm. Therefore, the efficacy of this method for patients with large segmental bone defects remains unproven.

With advancements in 3D printing and foundational research in engineering, 3D printing technology holds great promise as an alternative to allograft reconstruction, supporting early functional exercise while reducing complications associated with biological reconstructions [41]. In orthopedic applications, 3D printing offers significant advantages in areas, such as weight-bearing capacity, tissue regeneration, and initial stability. Metals, like titanium and cobalt-chromium alloys, are commonly used in load-bearing implants due to their excellent mechanical stability [42-44]. Additionally, 3D-printed models provide a detailed visualization of bone tumor morphology and its relation to surrounding anatomical structures, enabling precise surgical planning. 3D-printed metal prostheses also allow for rapid rehabilitation and early weight-bearing post-surgery. Hollander *et al*. studied the Ti-6Al-4V titanium alloy, using scanning electron microscopy on an *in vitro* porous model to show osteoblast growth along pore rims, forming circular structures, thus confirming the alloy’s viability as a biomaterial alternative [45].

In our case, the contact surface between the prosthesis and the host bone was coated with titania-sprayed trabecular bone, promoting osteointegration and biological fixation, while reducing reliance on cement fixation. Fabricated using selective laser melting (SLM), an additive manufacturing (AM) method, the prosthesis was made from TiAl6V4 titanium alloy, chosen for its biocompatibility, mechanical stability, and corrosion resistance. The alloy's highly porous structure was critical for facilitating bone integration.The quality control of the porous structure was crucial, with pore size regulated between 100-500 microns and porosity maintained between 40% and 60% to balance strength, flexibility, and an environment conducive to bone growth. The surface roughness was controlled using laser processing to promote cell adhesion without causing excessive friction.

The anterior tibial vessel and the common peroneal nerve were fully enveloped by the patient’s tumor. In accordance with the principle of extensive resection, the tumor, along with the invading nerves and vessels, was removed. To mitigate the risk of potential foot drop as a complication, suture fixation was performed on the insertion of the foot extensor tendon and residual muscle tissue. Although the resection of the patient’s tibia involved a lengthy tumor segment, the lower boundary of the tumor was approximately 80 mm away from the distal tibial joint, sparing it from involvement. A full resection with an ankle fusion prosthesis could result in complications, such as prosthesis loosening or fracture, due to its length. Therefore, a prosthesis to preserve the ankle joint was selected to retain sufficient distal tibial structure. Distal plates and screws were employed to reinforce both the prosthesis and the limb, while the remaining fibula was secured to the distal tibia using metal cables to reconstruct the lower tibiofibular syndesmosis, thereby enhancing stability and promoting biological healing. To treat foot drop resulting from injury to the common peroneal nerve, Fahmy *et al*. utilized two hollow rivets to secure the talofibular joint, following the cleaning of the articular cartilage of the talus and tibia through ankle arthroscopy [46]. Ankle joint fusion can reduce stress on joint mobility and may facilitate faster recovery. However, complications may arise, including talus prosthesis loosening and fretting wear between the prosthesis and the articular surface due to lower limb stress imbalance [47]. Additionally, reconstructing the ligaments presents challenges. The tibial nerve is considered a suitable donor for restoring the function of the tibialis anterior muscle, and dorsiflexion of the ankle joint can be reconstructed through partial tibial nerve transplantation [48]. In addition to nerve transfer, tendon transposition is also an effective method for enhancing joint independence and mobility. Stevoska *et al*. conducted a systematic review of tendon transfer techniques for foot drop, analyzing a total of 44 cohort studies [49]. Among these studies, 33 utilized posterior tibial tendon transfer *via* the interosseous membrane, 10 *via* the peritibial pathway, and 1 used gastrocnemius tendon transfer. After various follow-up periods, 94% of patients (205 out of 219) reported satisfaction with the outcome of the operation, with 29 patients indicating an ankle range of motion between 10° and 40° for both plantar flexion and dorsiflexion. Based on the patient’s requirements and actual conditions, the tendon and surrounding muscles were reinforced with sutures rather than undergoing ankle fusion or transposition. The patient was advised to use a cast to immobilize the ankle joint in a dorsiflexion-neutral position for 6 to 8 weeks. If foot drop persists, consideration may be given to ankle fusion surgery.

## 4 LIMITATIONS

Although the 6 to 12-month follow-up enabled us to observe the integration of the prosthesis and the recovery of joint function, we recognize that these evaluations were short-term. The relatively brief follow-up period limited our ability to assess the long-term durability of the customized 3D-printed prosthesis and potential complications. Therefore, a long-term follow-up plan has been developed for the patient, with annual assessments involving clinical evaluation, radiological imaging, and functional testing. These visits will be crucial for monitoring the patient's progress and identifying any potential long-term complications, such as prosthesis loosening, joint dysfunction, or tumor recurrence. Additionally, the single case presented in this study limits the generalizability of the findings. Future studies with larger sample sizes and extended follow-up periods are needed to further explore the efficacy and safety of this approach in a broader patient population.

## CONCLUSION

This case highlights the successful use of a customized 3D-printed prosthesis for the reconstruction of a critical-sized tibial defect following the resection of a myxofibrosarcoma (MFS) in the lower leg. The individualized prosthesis preserved both the knee and ankle joints, offering a promising solution for patients with large, complex tumors involving critical bone structures. Early postoperative follow-up showed satisfactory functional outcomes, including preserved joint motion and no signs of recurrence or metastasis.

## Figures and Tables

**Table 1:** Reported cases of MFS in the lower leg over the past 20 years.

Refs.	**Case Number**	**Gender/Age**	** Recurrent Case (Yes/No)**	**Site (size)**	** Infiltrated Structure**	**Histological Analysis**	**Immunohistochemical Stains**	**Primary Treatment**	**Other Treatments**	**Follow-up\Outcomes **
Payal *et al* [7]	1	31,M	No	middle and distal thirds of the anterior cortex of left tibia(5 cm in maximum dimension)	the surrounding adipose tissue and cortical bone	LG MFS	—	local excision	—	6m\no evidence of recurrence
Marotta *et al* [5]	1	84,M	No	The anteromedial side of tibial mid-diaphysis (8 × 5 cm))	No	HG MFS	CD34(-),SMA(-), desmin(-), keratins(-), caldesmon(-), S-100(-)	Wide resection, followed by reconstruction with cement, plate and a musculocutaneous gastrocnemius flap	postoperative radioterapy	2y\good functional outcome, no recurrence and metastasis
Paul *et al* [8]	6	46-84, 4F/2M	No	calf (1.0-17.0cm average 8.6cm)	—	1 LG MFS 1 IG MGS 4 HG MFS	1 case vimentin(+), CD34(+), CD99(part+); SMA(-), desmin(-), TLE1(-), S100(-), EMA(-), MUC4(-);1case vimentin(+) CD68(+); desmin(-), AE1/AE3(-), CD1a(-), HMB45(-), SOX10(-), CD117(-), S100(-), EMA(-), SMA(-), TFE3(-), CD31(-), CD45(-), PAX5(-), lysozyme(-)	—	—	—
Ahmed *et al* [4]	1	50,F	No	the medial aspect of left leg (20x20x25 cm)	the medical aspect of gastrocnemius and soleus	HG MFS	ASMA(+), CD34(+)	Wide excision	postoperative radiotherapy	no evidence of metastasis.
Teresa *et al* [3]	1	68,M	No	the lower left distal leg(8 ×6 cm)	distal fibula, distal tibiofibular joint and the lateral cortex of the distal tibia	HG MFS	—	Wide resection of the tumor and ankle joint salvage reconstruction with an ipsilateral fibula free flap	postoperative chemo and radiotherapy	3y\complete ROM of ankle joint, tumor free, no complications
Esaki *et al* [6]	1	88,F	Yes	distal left lower leg (6 × 4 × 12 cm)	No	MFS	—	Hyperthermia and radiotherapy	Local excision of the recurrenced mass	2.5y\Wound infection, no recurrence or metastases

**Table 2:** Tabular comparison of biological *vs*. mechanical reconstruction methods.

**Method**	**Efficacy**	**Complications**	**Application Scope**	**Limitations**
**Allografts**	Gold standard for tumor reconstruction. Bone conduction and induction are promoted.	High risk of fracture, infection, nonunion. Limited availability of large bone grafts.	Used for **medium to large bone defects** (*e.g*., hip, knee).	Immune rejection, infection, and difficulty in obtaining large segments.
**Vascularized Fibula Grafts (VFG)**	Effective for smaller defects. Provides bone regeneration and stability.	Fatigue fractures, complications at donor site (*e.g*., compartment syndrome).	Used for **medium-sized defects**, especially in **weight-bearing bones** like tibia.	Not suitable for large defects due to weakness. Requires stable vascular supply.
**Autografts (Tumor-Devitalized)**	Can provide biological fixation if successful. Utilizes patient's own tissue, reducing rejection.	Increased operation time, nonunion, delayed union, infection, weight-bearing difficulties.	Used for **small to medium defects** where **self-tissue** can be used.	Devitalization process may result in complications, leading to delayed healing.
**3D-Printed Custom Prostheses**	Effective for “Ultra-Critical Sized Bone Defects.” High precision and custom fit. Allows for rapid rehabilitation and early weight-bearing.	Early bone integration challenges, soft tissue complications.	Used for **large, complex defects**, especially those exceeding 15 cm.	Expensive, requires advanced surgical techniques. Long-term success depends on host bone integration.

## Data Availability

The data relevant to this study will be available on request from the corresponding authors [J.Z] and [J.W].

## LIST OF ABBREVIATIONS

MFS: Myxofibrosarcoma
3D: Three-dimensional
MRI: Magnetic resonance imaging
MSTS: Musculoskeletal Tumor Society
STSs: Soft tissue sarcomas
nRT: Neoadjuvant radiotherapy
LSS: Limb-sparing surgery
VFGs: Vascularized fibula autografts

## STANDARDS OF REPORTING

CARE guidelines were followed.
