# Supplementary material for: Reconstruction with a Custom-made 3D-printed Prosthesis for Limb Salvage and Joint Preservation after Resection of the Calf Myxofibrosarcoma Involving the Tibia: A Case Report and Literature Review
Source: Curr Med Imaging. 2025 Oct 29;22:e15734056415924. doi: 10.2174/0115734056415924251022041623 (PMC13463226; doi:10.2174/0115734056415924251022041623)
Supplement: Supplementary file 1 [file CMIM-22-E15734056415924_SD1.pdf]

1573-4056/25 *Send Orders for Reprints to reprints@benthamscience.net*

Current Medical Imaging

Content list available at: https://benthamscience.com/journals/cmir

Supplementary Material

Reconstruction with a Custom-made 3D-printed Prosthesis for Limb Salvage and Joint Preservation after Resection of the Calf Myxofibrosarcoma Involving the Tibia: A Case Report and Literature Review

Haoyuan Du[1], Ping Wang[3], Qi Liu[1], Zhiping Deng[4], Weifeng Liu[4], Jie Zhao[2,4,*] and Junqiang Wang[1,*]

[1]*Department of Orthopaedic Surgery, Beijing Jishuitan Hospital, Capital Medical University, Beijing, 100035, China*
[2]*Department of Orthopaedic Surgery, The Affiliated Hospital of Shandong University of Traditional Chinese Medicine, Jinan, Shandong 250014, China*
[3]*Department of Radiology, Beijing Jishuitan Hospital, Capital Medical University, Beijing, 100035, China*
[4]*Department of Orthopaedic Oncology Surgery, Beijing Jishuitan Hospital, Capital Medical University, Beijing, 100035, China*

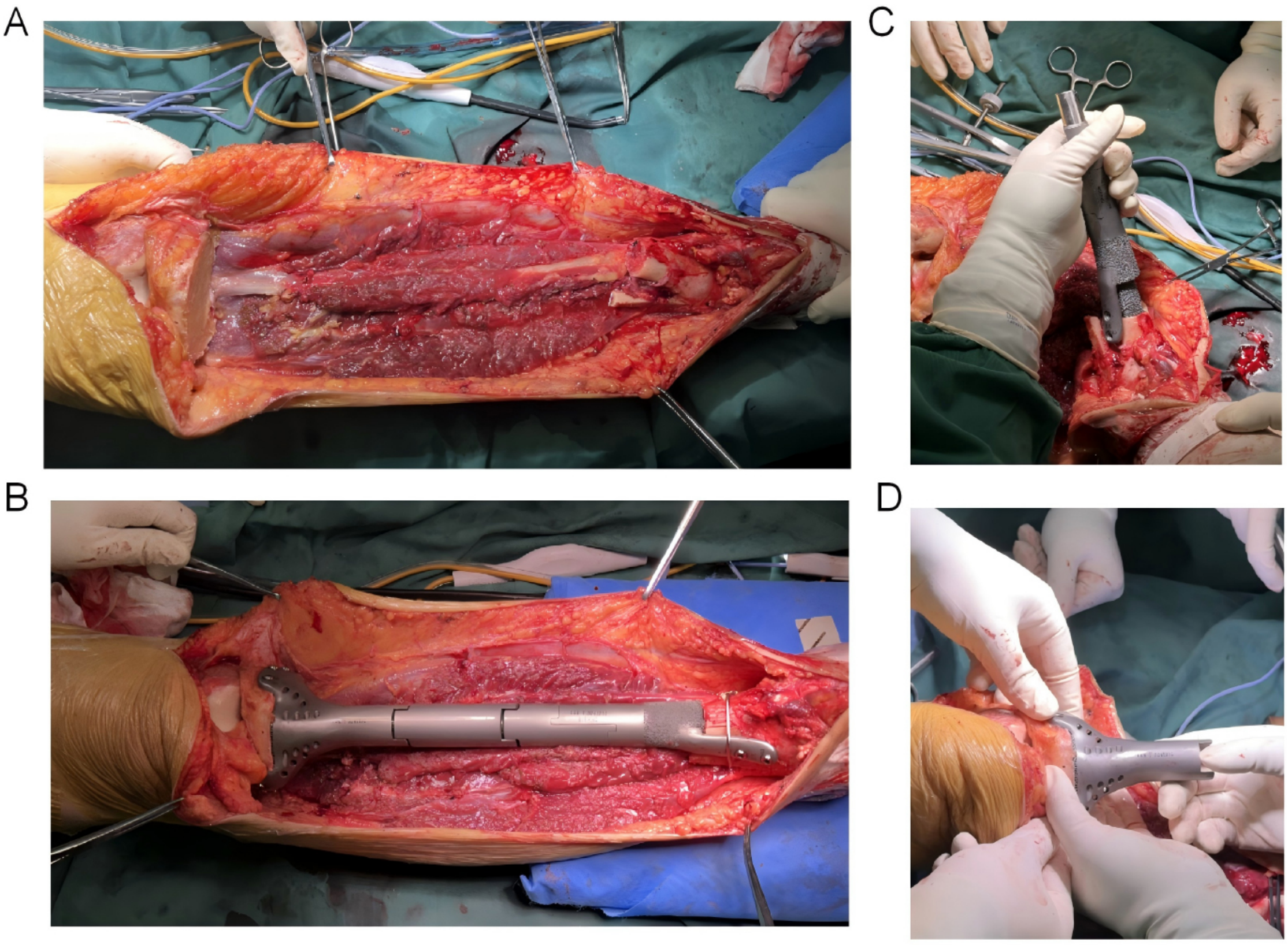

Fig. (S1). (**A**) Soft tissue and bone of the residual calf after osteotomy assisted by a 3D osteotomy guide plate. (**B**) Intraoperative placement of a distal tibial prosthesis with plate and screws on the outer side to enhance the joint. (**C**) After implantation of the 3D printed prosthesis, the titanium cables were used to bind and fix the residual tibia and fibula. (**D**) Intraoperative placement of the proximal tibial prosthesis with screws on bilateral sides.

DOI: 10.2174/0115734056415924251022041623, 2025, *21*, e15734056415924

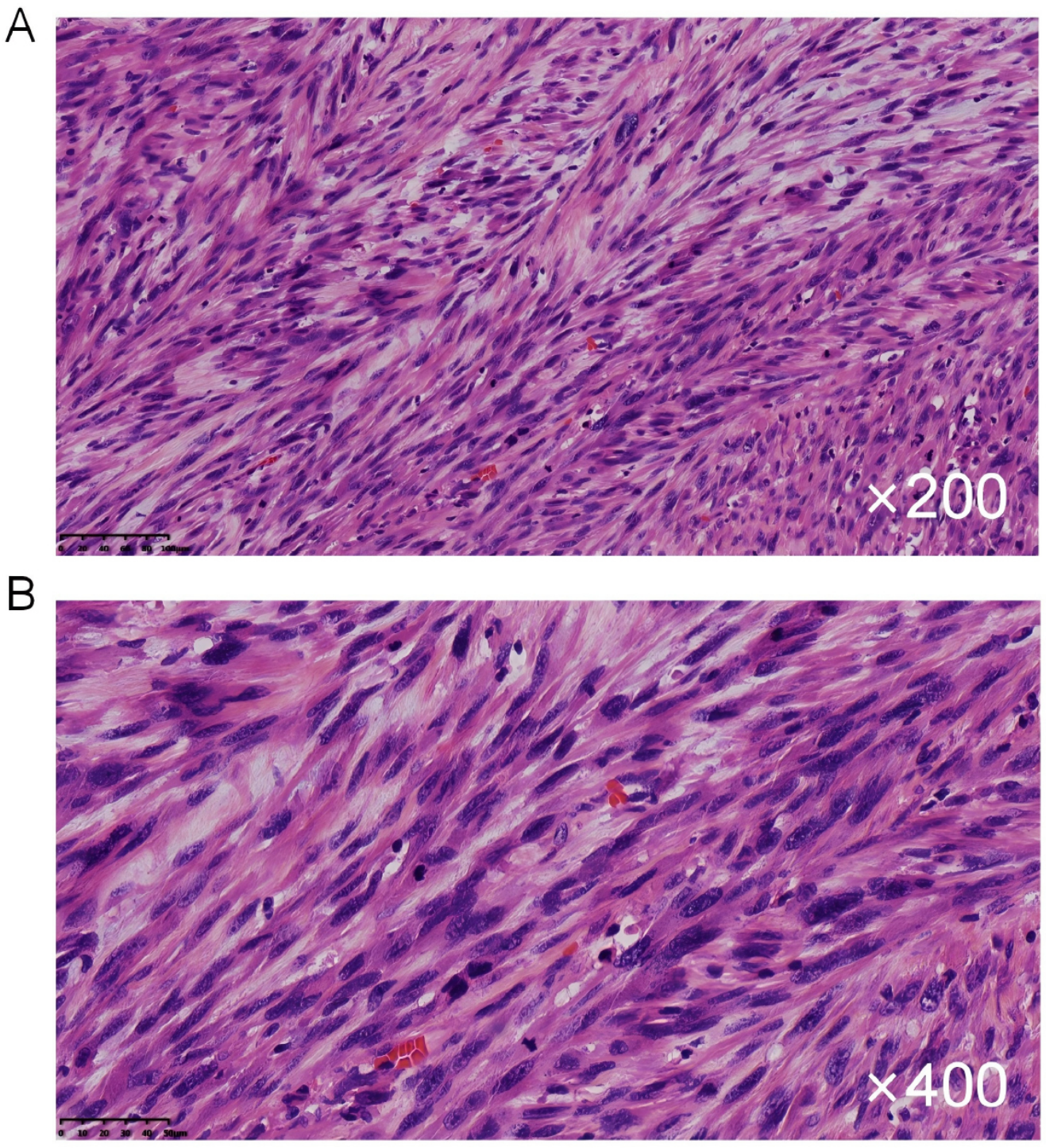

Fig. (S2). Microscopic image of the lesion with hematoxylin-eosin staining. (**A**) The low-power image shows the tumor with myxoid stroma. (**B**) The high-power view demonstrates the cells to be epithelioid with abundant eosinophilic cytoplasm and more pleomorphism, and abundant mitotic figures.

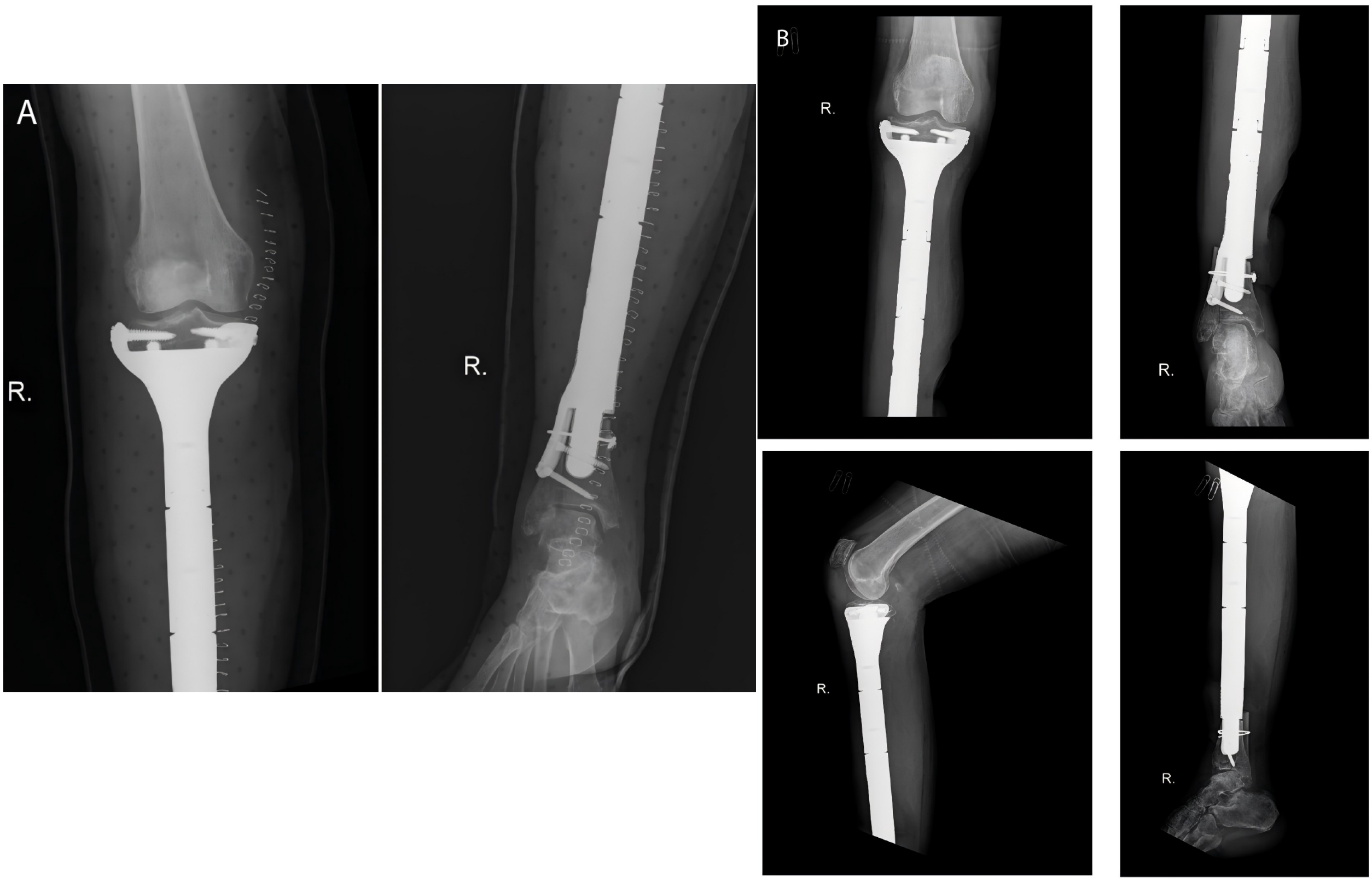

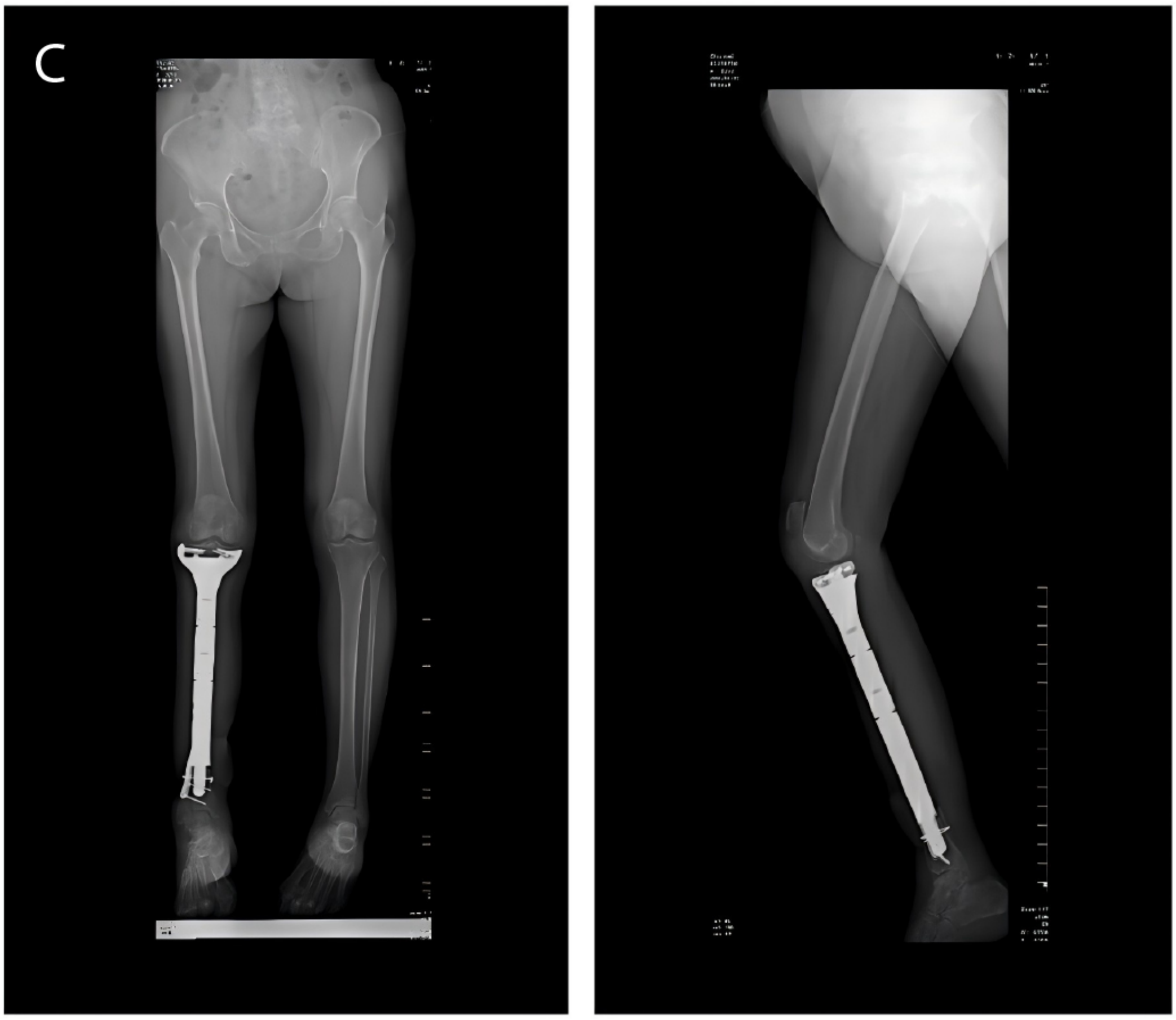

Fig. (S3). At 2 weeks after the operation, radiography images showed no obvious loose zones around the prosthesis stem, knee joint, and malleolus.

© 2025 The Author(s). Published by Bentham Science Publisher.

This is an open access article distributed under the terms of the Creative Commons Attribution 4.0 International Public License (CC-BY 4.0), a copy of which is available at: https://creativecommons.org/licenses/by/4.0/legalcode. This license permits unrestricted use, distribution, and reproduction in any medium, provided the original author and source are credited.
